# New Approaches on the Anti-Inflammatory and Cardioprotective Properties of *Taraxacum officinale* Tincture

**DOI:** 10.3390/ph16030358

**Published:** 2023-02-26

**Authors:** Alexandra Epure, Alina E. Pârvu, Laurian Vlase, Daniela Benedec, Daniela Hanganu, Ovidiu Oniga, Ana-Maria Vlase, Irina Ielciu, Anca Toiu, Ilioara Oniga

**Affiliations:** 1 Department of Pharmacognosy, Faculty of Pharmacy, “Iuliu Hațieganu” University of Medicine and Pharmacy, 8 V. Babeș Street, 400012 Cluj-Napoca, Romania; 2 Department of Physiopathology, Faculty of Medicine, “Iuliu Hațieganu” University of Medicine and Pharmacy, 8 V. Babeș Street, 400012 Cluj-Napoca, Romania; 3 Department of Pharmaceutical Technology and Biopharmacy, “Iuliu Hațieganu” University of Medicine and Pharmacy, 8 V. Babeș Street, 400012 Cluj-Napoca, Romania; 4 Department of Pharmaceutical Chemistry, Faculty of Pharmacy, “Iuliu Hațieganu” University of Medicine and Pharmacy, 8 V. Babeș Street, 400012 Cluj-Napoca, Romania; 5 Department of Pharmaceutical Botany, Faculty of Pharmacy, “Iuliu Hațieganu” University of Medicine and Pharmacy, 23 Gheorghe Marinescu Street, 400337 Cluj-Napoca, Romania

**Keywords:** *Taraxacum officinale*, cardioprotective, anti-inflammatory, antioxidant, polyphenols, cichoric acid

## Abstract

The present research investigated the in vivo anti-inflammatory and cardioprotective activities, as well as the antioxidant potential of *Taraxacum officinale* tincture (TOT), in relation to the polyphenolic composition. Chromatographic and spectrophotometric techniques were used to determine the polyphenolic profile of TOT and the antioxidant activity was preliminarily assessed in vitro by DPPH• and FRAP spectrophotometric methods. The in vivo anti-inflammatory and cardioprotective activities were studied in rat turpentine-induced inflammation and in rat isoprenaline-induced myocardial infarction (MI) models. The main polyphenolic compound identified in TOT was cichoric acid. The oxidative stress determinations showed the capacity of the dandelion tincture not only to decrease the total oxidative stress (TOS), the oxidative stress index (OSI), and the total antioxidant capacity (TAC), but also the malondialdehide (MDA), thiols (SH), and nitrites/nitrates (NOx) levels both in inflammation and MI models. In addition, aspartate aminotransferase (AST), alanine aminotransferase (ALT), creatin kinase-MB (CK-MB), and nuclear factor kappa B (NF-κB) parameters were decreased by the administration of the tincture. The results show that *T. officinale* could be considered a valuable source of natural compounds with important benefits in pathologies linked to oxidative stress.

## 1. Introduction

Cardiovascular diseases are the main cause of global disability, and health predictions show that they will also be the most prominent cause of death in 2030. CVDs include myocardial infarction (MI), congestive heart failure, coronary heart disease angina, and peripheral arterial disease [1].

MI can be mediated via several biochemical mechanisms, such as reactive oxygen species (ROS), defective antioxidant enzymes, oxidative stress, and inflammatory process. In pathologic conditions, the disproportion between ROS and antioxidants promotes myocardial cell damage, necrosis, and apoptosis [2]. Therefore, it is necessary to improve the myocardial redox status by protecting the antioxidants and stabilizing the oxidants. Several factors are crucial in the progression of MI and reperfusion injury: oxidative stress, induction of inflammation, inflammatory cell infiltration, and activation of adaptive immune response. Therefore, using agents that can reduce their pathological mechanisms during MI and reperfusion injury by regulating these pathological mediators is a priority [3].

*Taraxacum officinale* (L.) Weber ex F.H.Wigg. (dandelion) is a perennial herbaceous flowering species of the Asteraceae family [4]. The chemical composition consists of phenolic compounds (polyphenolic acids, flavonoids, coumarins, tannins), sesquiterpene lactones (taraxacin, lactucopicrin, and cichorin, found mostly in the roots), triterpenes (α-amyrin, β-amyrin, lupeol, taraxol, taraxasterol), sterols (stigmasterol, β-sitosterol), polysaccharides (especially inulin in roots), minerals, amino acids, and vitamins that can be found in all the organs of the species [5]. Several hydroxybenzoic acids (protocatechuic, vanillic, syringic and gallic acids), hydroxycinnamic acids (*p*-coumaric, caffeic, ferulic, and synapic acids), and derivatives (chlorogenic, caftaric, cichoric acids) were identified in *T. officinale* [6], and cichoric acid was the main compound found in the aerial parts [7]. Dandelion also contains flavonoids, such as quercetin, kaempferol, apigenin, luteolin, catechins, hyperoside, isoquercitrin, quercitrin, rutin [8]. *T. officinale* has important pharmacological effects: antioxidant (aqueous extracts of *Taraxaci herba sin flos*, *T. radix*, *T. flos*), antihyperglycemic (ethanolic extracts *T. radix*), cholagogue (methanolic extracts *T. folium*), diuretic (aqueous extracts *T. herba*, *T. radix*), anti-inflammatory (methanolic etracts, *T. flos*), immunomodulatory and anti-allergic (isolated compounds from *T. officinale*), anti-thrombotic (ethanolic extracts of *T. radix*) and prebiotic (aqueous extracts of *T. radix*) [4].

The in vivo hypolipidemic, anti-obesity, and hepatoprotective properties of *T. officinale* extracts are linked to the antioxidant activity, due to the rich polyphenolic composition that may correct oxidative stress, with positive outcomes in numerous diseases (chronic inflammations, neurodegenerative disorders and metabolic syndrome) [2]. Additionally, the effects of the polyphenols on the cardiovascular system are based on mechanisms such as antihypertensive, anti-atherosclerotic, and anti-inflammatory effects; improving the lipid profile; and a direct effect on endothelial cells [9].

Several studies suggested that *T. officinale* presented valuable anti-inflammatory potential [10] and various in vivo studies evaluated the hypolipidemic properties of lowering hypertension and decreasing lipid peroxidation [11]. The aim of the present study is the in vivo evaluation of anti-inflammatory and cardioprotective effects of *T. officinale* tincture (TOT) based on the antioxidant mechanism of the contained compounds. The novelty of the present study consists of the fact that, to the best of our knowledge, it represents the first report on the TOT cardioprotective activity evaluated in vivo on an isoprenaline-induced myocardial infarction model. Moreover, the present study aims to highlight the in vivo anti-inflammatory activity of TOT. 

## 2. Results and Discussion

### 2.1. Total Polyphenolic Content (TPC), Total Flavonoidic Content (TFC), and Total Caffeic Acid Derivatives Content (TCADC)

The results obtained by the spectrophotometric analysis of polyphenols from TOT are presented in Table 1.

The TPC in TOT was comparable with some of the data reported by other authors (18.53 mg GAE/g d.w., for 20% ethanolic extract; 33.53 mg GAE/g d.w., for 40% ethanolic extract; 33.90 mg GAE/g d.w., for aqueous ethanol extract 1:1) [12]. Other researchers reported different values for TPC from *T. herba*, depending on the extraction conditions and solvents: 123.42 mg GAE/g d.w. (for 60% ethanolic extract); 70.46 mg GAE/g d.w. (for 80% ethanolic extract); 41.47–691.6 mg GAE/g d.w. for aqueous and hydroalcoholic extracts from Pakistan; 33.94 mg GAE/g d.w. (for 1:1 aqueous ethanolic extract) and 23.27 mg GAE/g d.w. (for 80% ethanolic extract, plants harvested from USA) [12]. 

Regarding the TFC, TOT was characterised by lower values than those reported in USA as 14.00 mg RE/g d.w. for aqueous ethanolic extracts 1:1 and 12.35 mg RE/g d.w. for 80% ethanol extracts, and also by those reported for dandelion aerial parts gathered in Malaysia with values ranging from 12.82 to 55.81 mg RE/g d.w for 20 to 80% ethanol extracts [12,13]. Differences could be determined by the particularities of the analytical method and the conditions of obtaining the raw material and extracts.

To the best of our knowledge, the TCADC content in TOT was not reported before and it was evaluated in this study for the first time.

Our experiments showed that *T. officinale* harvested from Romania is a raw material rich in polyphenols, with important content of phenol acids derivatives and flavonoids.

### 2.2. HPLC-UV-MS Analysis

In order to characterise the TOT, HPLC-UV-MS analyses were conducted in several stages, aiming to identify and quantify polyphenolic compounds. In the first stage, phenolic acids with highly hydrophilic character were determined, then other polyphenolic compounds, and in the end, cichoric acid was identified and quantified (Table 2).

The chromatograms are available in Supplementary Material (Figures S1–S3).

Cichoric acid (2, 3 dicaffeoyl-tartaric acid) was the main polyphenolic compound identified in TOT. These results are in accordance with current data that shows *T. officinale* as a source of cichoric acid [14].

Different amounts, between 0.9 and 9.2 μg/g d.w. were determined in TOT for syringic, vanillic, and protocatechuic acids, while ferulic acid was present in higher concentration (53.60 μg/g d.w.). Among the flavonoids, quercetin-O-glycosides, such as quercitrin (present in higher quantity) and rutin were identified and quantified, together with free aglycons, luteolin, and apigenin, in quantities between 5–44 μg/g d.w. In dandelion extracts analysed by Xue et al., cichoric acid was present at a quantity of 24,031 µg/g d.w. for 50% ethanol extract, consistent to our TOT HPLC analysis. Generally, the concentrations of some phenolic acids and flavonoids were comparable to our findings [13]. Previous analysis of our team quantified cichoric acid at a value of 7163.11 µg/g d.w. for a tincture, prepared with the vegetable material gathered from a lower altitude, a grassland region, characterised by a dry climate with higher temperatures and lower levels of precipitation [15]. The material analysed in the present study was harvested from a plateau area, a higher altitude distinguished by moderate temperatures and higher levels of precipitation. The differences could be explained by the influence of the pedo-climatic conditions on the biosynthesis of the compounds in plants. 

Cichoric acid has been described to exhibit many pharmacological activities with benefits in various pathologies. Some properties such as antimicrobial, anti-inflammatory, anti-tumoral, anti-diabetic, neuroprotective, and hyaluronidase inhibiting activities, among others, are reported [14,16].

Based on the current research, the *T. officinale* aerial parts could also be considered a valuable source of polyphenols such as cichoric acid.

### 2.3. Antioxidant Activity 

The in vitro antioxidant activity of TOT was evaluated using two assays: DPPH• and FRAP. The results obtained for the antioxidant capacity determinations are summarised in Table 3.

For the DPPH assay, the EC50 value has an opposite relation with the antioxidant capacity; a greater antioxidant capacity is achieved with the decrease of EC50 value. 

The assessment of antioxidant activity by the FRAP method revealed a good antioxidant capacity of TOT, comparable with some others’ reports. The data from the literature shows very different values that quantify the antioxidant activity of dandelion extracts by the FRAP method, ranging between 3.32 and 131.5 μM TE/g d.w., using different concentrations of extracts (40%, 70%, and 96% ethanolic extracts of *T. herba* from plant material harvested in Bulgaria and Poland) [17,18].

Our results for the in vitro antioxidant activity evaluation were in agreement with the TPC of the tested extracts.

The current knowledge regarding the relationship between polyphenols and antioxidant activity hints that the antioxidant activity of *T. officinale* extract is associated with the quantity of total phenolic acids, which was higher than flavonoids content in our tincture [13]. 

### 2.4. Pharmacological Studies

#### 2.4.1. The Evaluation of In Vivo Anti-Inflammatory Effects 

Three concentrations (100, 50, and 25 mg/mL) of the TOT sample (namely TOT 100, TOT 50, and TOT 25, respectively) were administrated on rats in a turpentine oil-induced acute inflammation in vivo experiment. To assess the effects, serum oxidative stress markers were analysed. The results are summarised in Table 4.

The experiment evaluated the oxidative stress through general tests such as TOS, OSI, and TAC and by specific tests as MDA, SH, and Nox. In addition, the NF-κB parameter, which has an important role in pro-inflammatory cytokines signalling, was assessed. 

Turpentine oil is an inflammatory catalytic agent that activates phagocytes by boosting the Nox and ROS species production [19]. When an inflammation process is triggered, TOS, and subsequently, OSI parameters manifest increased values comparative to the healthy animals, as seen in our experiment (*p* < 0.001). Additionally, the same pattern applies to Nox and MDA levels whilst the TAC and SH levels decrease (*p* < 0.001). NF-κB controls the transcription of pro-inflammatory cytokines among other substances mediating inflammatory responses (NF-κB signalling in inflammation), thus, a current inflammation status is correlated with higher NF-κB concentrations (*p* < 0.05). All three TOT samples lowered TOS and OSI parameters (*p* < 0.001). The Nox levels were improved by TOT 25 (*p* < 0.01), and TOT 100 improved both SH (0.01) and NF-κB levels (*p* < 0.05). The TAC and MDA concentrations were not influenced by any TOT sample.

These findings show that TOT has an antioxidant effect in rats’ turpentine-induced inflammation by correcting the oxidant levels and increasing the antioxidants. Furthermore, TOT has a direct anti-inflammatory effect by reducing NF-κB levels. In this study, the non-steroidal anti-inflammatory diclofenac was used as a positive control. The oxidative parameters for the animals that received the drug were not significantly modified (*p* > 0.05), but the NF-κB parameters were lowered (*p* < 0.05). This is in accordance with the drug’s pharmacological mechanism, which involves the nonselective inhibition of cyclooxygenase enzymes (COX 1 and COX 2), thus inhibiting prostaglandin synthesis—molecules that play a key role in inflammation and pain [20]. We compared the results determined by TOT for NF-κB with the diclofenac group, and there were no significant differences (*p* > 0.05) suggesting that TOT has a comparable effect with diclofenac regarding the NF-κB status. 

The obtained results highlight the fact that the treatment with TOT on rats with turpentine-induced inflammation, determined anti-inflammatory effects by reducing NF-κB expression and by reducing oxidants. 

**Figure 1:**
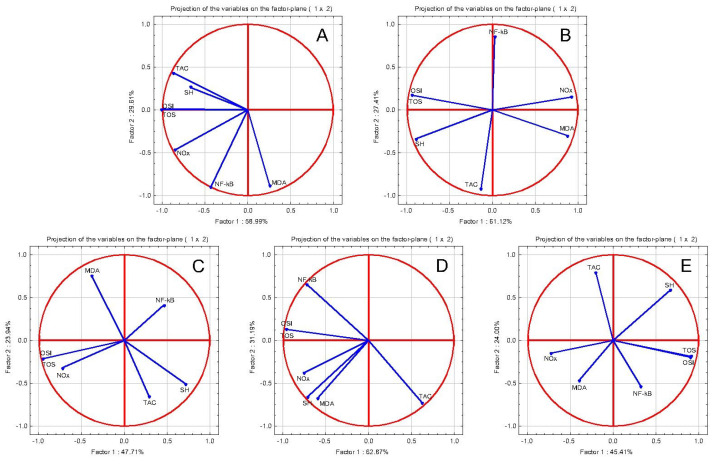
Anti-inflammatory oxidative stress tests PCA results: PCA correlation circles in turpentine oil-induced acute inflammation: (**A**) INFLAMMATION group: NF-kB increase was positively correlated with oxidants tests, specifically, TOS, OSI, Nox, and MDA (**B**) DICLOFENAC group: NF-kB reduction was positively correlated only with TOS, OSI, and Nox (**C**) TOT 100 group: NF-kB reduction was positively correlated only with MDA (**D**) TOT 50 group: NF-kB reduction was positively correlated only with TOS, OSI, and Nox (**E**) TOT 25 group: NF-kB reduction was positively correlated only with TOS, OSI, and MDA.

With the aim to assess the relationship between the parameters, a PCA correction circle was used (Figure 1). Original Pearson Correlation information is available in Supplementary Material (Tables S1–S5).

This type of representation involves grouping the different features according to their positive and negative correlation. Features with a positive correlation will be grouped together. The uncorrelated ones are orthogonal to each other, and negative correlations will be plotted on the opposing quadrants of the plot. We obtained the coordinates of the values by calculating the correlation between each original variable and their associated components [21].

In the tested groups: CONTROL, DICLOFENAC, TOT 100, TOT 50, and TOT 25, the oxidative stress parameters and the transcription factor NF-κB were overall correlated, concluding that inflammation was associated with oxidative stress. 

Several studies show that inflammation and oxidative stress are interdependent. In an outgoing inflammatory process, the phagocytic cells (macrophages and neutrophils) activate. This produces large amounts of reactive oxygen species and reactive nitrogen species, including hydrogen peroxide, hydroxyl free radical, superoxide, nitric oxide, and peroxynitrite [22,23].

Various molecular mechanisms involved in the anti-inflammatory activities of polyphenols include the inhibition of certain pro-inflammatory enzymes, such as ciclo-oxigenase 2 (COX2), lipo-oxigenase (LOX), and inducible nitric oxide synthase (iNOS). Polyphenols promote the inhibition of the transcription factor NF-kB and enhance the activation of a phase-II antioxidant enzymes, the mitogen-activated protein kinase, and protein kinase-C [9].

The anti-inflammatory effects of *T. officinale* extracts were evaluated in relationship with the polyphenolic composition and anti-oxidative effects. Jeon et al. describes the anti-inflammatory activity on lipopolysaccharides (LPS) which stimulated murine macrophage cells (RAW264.7) by improving NO, PGE2, and the cytokines TNF-α and IL-1 β levels, and inhibiting iNOS, COX2, and activation of MAP kinases, suggesting a direct anti-inflammatory effect in addition to the antioxidative mechanisms. Methanolic extracts of *T. officinale* also presented with NF-kB inhibition in an in vitro study conducted on LPSs in human umbilical vein endothelial cells [24]. 

Regarding in vivo models, aqueous extracts of *T. officinale* leaves lowered inflammation in cholecystokinin-induced acute pancreatitis in rats by blocking the production of IL-6 and TNF-α cytokines that depend upon NF-kB transcription [25]. Our TOT had an anti-inflammatory effect by inducing an important reduction of NF-kB.

In the air pouch model of carrageenan-induced inflammation, ethanolic extracts of *T. officinale* aerial parts inhibited the production of exudate and reduced the levels of leukocytes and nitric oxide within. It also exhibited a dose-dependent suppression on the vascular permeability of acetic acid abdominal induction assay in mice [10]. In turpentine oil- induced inflammation, TOT caused NOx reduction where the higher dilution had the strongest effect. Similarly, TOT lowered other oxidative stress markers, specifically, TOS, OSI, and MDA. For TOT, the anti-inflammatory activity was correlated with oxidative stress marker reduction (Figure 1). TOT had no significant activity on the antioxidant markers.

These results suggest that due to the phytochemicals present in *T. officinale*, TOT has an anti-inflammatory effect through inhibiting the activation of NF-κB and by lowering the oxidant concentrations. The phenolic compounds identified in *T. officinalis* could be involved in the anti-inflammatory effects of the extracts, as well as cichoric acid, based on its known antioxidant and anti-inflammatory properties [26]. Similar to cichoric acid, ferulic acid was found to exhibit anti-inflammatory effects, mainly due to the antioxidant properties [27]. The quantified flavonoids from our studied tincture, rutin and quercitrin, as well as luteolin and apigenin, showed anti-inflammatory effects in experimental tests [28]. Therefore, TOT anti-inflammatory activity based on the improvement of serum oxidative stress parameters could be associated with the presence of phenolic compounds with antioxidant properties, which could exhibit positive outcomes in inflammation treatment.

#### 2.4.2. The Evaluation of In Vivo Cardioprotective Effects 

TOT activity in turpentine-oil induced inflammation encouraged us to test the effect in the acute inflammation associated with the acute myocardial infarction (MI). In the present study, the TOT’s effect was evaluated on acute MI induced by isoprenaline. The parameters analysed in this experiment were NF-kB as an anti-inflammatory marker, TOS, OSI, TAC, MDA, SH, and NOx as oxidative stress markers, plus serum cardiac injury marker enzymes (AST, ALT, and CK-MB). The results are summarised in Table 5 and Table 6.

The consequences of MI induced by ISO were cell injury, indicated by the serum AST, ALT, and CK-MB increase, and an inflammatory response with elevated NF-κB. At the same time, an increased oxidative stress was indicated by the increased TOS, OSI, NOx, and MDA, and TAC and SH reduction.

In MI, all three TOT concentrations reduced myocardial cells injury enzymes, had anti-inflammatory activity by reducing NF-kB, and an antioxidant effect by lowering TOS, OSI, NOx, and MDA (*p* < 0.001). TOT 50 was the most effective concentration. SH was reduced after TOT treatments. A possible explanation for the decrease of SH levels is that in MI, prophylaxis by dietary antioxidant consumption reduces the formation of ROS and RNS and does not have an impact on preventing the reduction of previously present antioxidant species [29].

Overall, TOT 50 improved foremost the parameters (although there were no significant differences between the extract groups; *p* > 0.05) which is in accordance with the general knowledge that a mix of polyphenols in small doses can potentiate the antioxidant effect rather than single entities in higher doses; also at higher concentrations, some polyphenols can exhibit pro-oxidant effects via the Fenton reaction [30].

**Figure 2:**
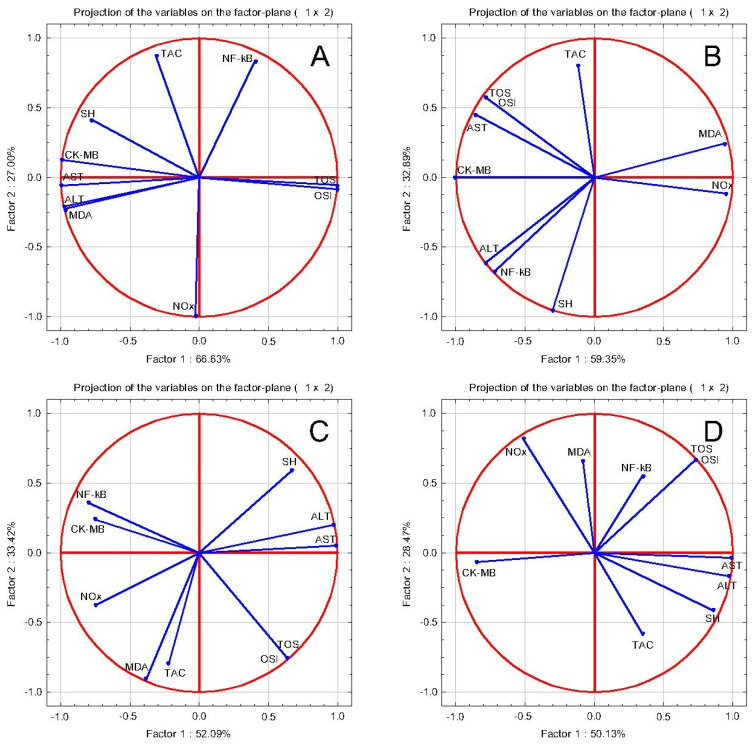
Anti-inflammatory, oxidative stress tests and cardiac function tests PCA correlation circles in isoprenaline-induced acute myocardial infarction: (**A**) ISO group: AST, ALT, and CK-MB correlate with MDA, and NF-κB with TOS and OSI; (**B**) TOT 100: AST, ALT, and CK-MB correlate with NF-κB, TOS, and OSI, and MDA with NOx; (**C**) TOT 50: AST and ALT correlate with TOS and OSI, CK-MB correlates with NOx and NF-κB; (**D**) TOT 25: AST and ALT correlate with TOS, OSI, and NF-κB, CK-MB correlates with NOx.

In order to evaluate the relationship between the parameters, a PCA was performed (Figure 2). Original Pearson Correlation information is available in Supplementary Material (Tables S6–S9). In general, in all groups there were correlations between AST, ALT, and CK-MB with the oxidative stress parameters, and also with NF-κB.

Cichoric acid, the major polyphenolic compound of *T. officinale* extract possesses cardioprotective activity, based on the improvement of cardiovascular homeostasis [31] and anti-atherosclerotic effect, based on antioxidative and anti-inflammatory mechanisms [32]. Ferulic acid also has cardioprotective effects [27].

The hydroxybenzoic acids (protocatechuic, vanillic, syringic acid) were studied in relation to cardiac and vascular applications, with positive outcomes [33,34].

Studies concerning flavonoids show positive outcomes in cardiovascular diseases, as rutin has an active role in reducing cardiac hypertrophy, and quercitrin (through its aglycon quercetin), has a role both as anti-atherosclerotic and as a cardiovascular risk improving agent [35,36]. Luteolin and apigenin exhibit cardioprotective effects with various applications in ischemia/reperfusion injury, atherosclerosis, and heart failure [36,37,38]. 

The heart is an organ that is vulnerable to oxidative stress because of the absence of antioxidant systems, therefore a systemic oxidative reduction may be favourable and diminish the myocardial injury during MI [39].

The presence of diverse polyphenolic compounds in TOT, such as phenolic acids and flavonoids with cardioprotective properties, may explain the capacity of the studied tincture to protect against MI induced by ISO, with a positive influence on serum oxidative stress parameters and serum cardiac injury markers. 

Other in vivo studies regarding the cardiovascular potential of *T. officinale* discuss the positive outcomes of extract administration in relation to metabolic syndrome (hypolipidemic effect by improving cholesterol and total lipids levels, and overall reduction in aortal thickness, as well as anti-obesity effects and an overall decrease in oxidative stress) and other disorders that can lead to heart damage [11]. The present study emphasises the positive influence of the active compounds from the tincture on some parameters involved in oxidative processes during the myocardium injury in MI.

Previous studies that compare the in vitro and in vivo antioxidant activities of different plant extracts show that these are not always correlated [39]. In the present experiment, TOT had a higher in vivo antioxidant activity compared to the in vitro antioxidant capacity, which was found to be good, using the described methods.

In the present study, we pointed out that TOT has an in vivo anti-inflammatory activity on turpentine-induced inflammation and cardioprotective effects on ISO-induced MI, by reducing the oxidative stress and inflammation. 

## 3. Materials and Methods

### 3.1. Chemicals and Reagents 

The references used in the LC-MS analysis were purchased from Sigma-Aldrich (Schnelldorf, Germany): cichoric acid (>95%), caffeic acid (≥98%), chlorogenic acid (≥95%), ferulic acid (>95%), sinapic acid (≥98%), hyperoside (≥98%), isoquercitrin (≥90%), quercitrin (quercetin 3-rhamnoside) (≥78%), quercetin (≥95%), luteolin (≥98%), kaempferol (≥97%), apigenin (≥95%), syringic acid (≥95%), protocatechuic acid (3,4-dihydroxybenzoic acid) (≥97%), and vanillic acid (≥97%). Ammonium acetate, acetonitrile, petroleum ether, chloroform, hydrochloric acid, acetic acid, potassium hydroxide, and Folin–Ciocâlteu reagent were purchased from Merck (Darmstadt, Germany). Sodium carbonate, sodium acetate trihydrate, and anhydrous aluminium chloride were purchased from Sigma-Aldrich (Schnelldorf, Germany). 2,4,6-tri(2-pyridyl)-1,3,5-triazine (TPTZ) reagent, 2,2-diphenyl-1-picryl-hydrazyl-hydrate (DPPH) reagent, and Trolox were acquired from Sigma-Aldrich (Schnelldorf, Germany); rutin (>95 %) was from Fluka Chemie GmbH (Buchs, Switzerland). Methanol p.a., ethanol 96%, and dichloromethane were purchased from Chemical Company (Iași, Romania) and iron chloride from Merck (Darmstadt, Germany). The commercial biochemistry kits for the pharmacological investigations (kit CK-MB-LQ. Anti CK-M. Immunoinh.; kit GOT/AST-LQ. IFCC. Enzymatic–UV; GPT/ALT-LQ. IFCC. Enzymatic–UV; kit UREA-LQ. Urease-GLDH. Kinetic; kit creatinine-J. J) were purchased from S.C. DG Diagnostics S.R.L. Cluj-Napoca. The turpentine used to induce inflammation was purchased from Sigma-Aldrich (Germany). Diclofenac sodium was purchased from a local pharmacy with the trademark Refen, concentration of 75 mg/mL, 3 mL (Hemofarm Koncern A.D.) Isoprenaline used to induce myocardial ischemia was purchased from Sigma-Aldrich. 

### 3.2. Plant Material and Extraction Procedure

*T. officinale* aerial parts (+*herba*) were harvested from Hunedoara County, (Lat. 45°45′18.151″ N/Long. 22°53′25.181″ E), Western Romania, during the flowering stage, from wild populations. A sample of the herbal material is available in the Pharmacognosy Department herbarium (voucher number 116). The plant material was air-dried, powdered, degreased with dichloromethane, and then was used for extraction. The tincture (1:10) was obtained from 50 g of plant material and 500 mL 70% ethanol, by maceration for 7 days at room temperature (*T. officinale* tincture = TOT) [39].

TOT was used for phytochemical analysis: total polyphenols content, total flavonoids content, and total caffeic acid derivatives content were determined. Then, HPLC-MS analysis, the antioxidant assays (DPPH• and FRAP methods), and the pharmacological experiments were performed using this tincture.

For the assessment of the anti-inflammatory and cardioprotective activities, *T. officinale* tincture (TOT 100, corresponding to 1 mg dry weight plant material/10 mL) was used, as well as two dilutions of the tincture, obtained with distilled water, TOT 50 (0.5 mg dry weight plant material/10 mL), and TOT 25 (0.25 mg dry weight plant material/10 mL).

### 3.3. Total Polyhenols Content Determination 

The total polyphenols content (TPC) was determined using the Folin–Ciocâlteu spectrophotometric method [40]. Briefly, TOT (2 mL) was diluted in a 25 mL volumetric flask with the same solvent and then, to 2 mL of each solution, 1 mL Folin–Ciocâlteu reagent and 10 mL of distilled water were added; the mixture was diluted to 25 mL with a solution of sodium carbonate (290 g/L). After 30 min in darkness, the absorbance of the samples was measured at 760 nm using a Cary 60 UV–Vis spectrophotometer from Agilent Technologies (R^2^ = 0.999). TPC is expressed as mg gallic acid equivalents (GAE)/g d.w. [15].

### 3.4. Total Flavonoids Content Determination

The total flavonoids content (TFC) of TOT was performed by a spectrophotometric method based on the colour reaction with AlCl_3_ reagent. Briefly, 10 mL of the TOT was diluted to 25 mL using methanol. To 5 mL of the diluted solution, 5.0 mL sodium acetate (100 g/L) and 3.0 mL aluminium chloride (25 g/L) were added; the obtained solution was diluted with methanol up to 25 mL in a calibrated flask. The absorbance was measured at 430 nm and the results are expressed as mg rutin equivalents (RE)/g d.w. (R^2^ = 0.999) [40]

### 3.5. Total Caffeic Acid Derivates Content Determination

The caffeic acid derivates content (TCADC) of the TOT was analysed using Arnow reagent by a spectrophotometric method. In short, in a volumetric flask, 10 mL of the TOT was mixed with methanol at 25 mL, and 5 mL of the obtained solution was diluted with ethanol 50% at 10 mL. Next, 1 mL of HCl 0.5 N, was added to 1 mL of solution, together with 1 mL of Arnow reagent and 1 mL NaOH 1 N; the mixture was increased to 10 mL with ethanol 50% [41]. The absorbance (λ = 500 nm) was used to calculate the results, expressed as mg caffeic acid equivalents (CAE)/g d.w. (R^2^ = 0.994). 

All phytochemical determinations were performed in triplicate.

### 3.6. Evaluation of the In Vitro Antioxidant Capacity

#### 3.6.1. DPPH Radical Scavenging Activity

The antioxidant activity was assessed in vitro by different methods. The assay measures the scavenging ability of extracts towards DPPH, a stable nitrogen cantered free radical. First, 2 mL of TOT (in different concentrations) were added to 2 mL of 0.1 g/L DPPH• methanol solution. The absorbance was determined at 517 nm, after 30 min of incubation at room temperature in the dark (R^2^ = 0.997). The DPPH radical scavenging activity (AA) was calculated as follows: AA% = (A_control_ − A_sample_/A_control_) × 100, where A_control_ is the absorbance of DPPH• radical + methanol (does not contain the sample) and A_sample_ is the absorbance of DPPH• radical + sample extract. The EC50 (µg/mL) values were also calculated in order to determine the half maximal inhibitory concentration of TOT [42,43]. The experiments were performed in triplicate.

#### 3.6.2. Ferric-Reducing Antioxidant Power Assay

This assay has been proven to measure the antioxidant capacity of plant products containing polyphenols. The absorbance was measured at 450 nm. A volume of 0.4 mL of TOT was diluted with water to 1.8 mL and mixed with 6 mL of FRAP reagent (R^2^ = 0.992). The antioxidant activity was expressed as Trolox equivalents (TE) [44]. All determinations were realised in triplicate.

### 3.7. HPLC-UV-MS Separation

The phytochemical assay of the tincture was performed by liquid chromatography coupled with mass spectrometry (Agilent Technologies 1100 HPLC Series system coupled to an Agilent 1100 mass spectrometer (LC/MSD Ion Trap SL)). The separation was achieved using a reverse-phase analytical column (Zorbax SB-C18 100 × 3.0 mm i.d., 3.5 μm particles, t° = 48 °C). The injection volume was 5 µL and the flow rate was set to 1 mL/min. The MS system functioned using an electro spray ion source in negative mode. Chromatographic data have been interpreted using ChemStation and Data Analysis. Compound identification was performed in both UV and MS mode, by comparing their traces/spectra obtained in the experiment with spectra from the data library. The limit of detection was 0.1 μg/mL and the limit of quantification was 0.5 μg/mL. The UV trace was used for quantification of identified compounds from MS detection. Quantitative determinations were performed using an external standard method. Calibration curves in the 0.5–50 μg/mL range with good linearity (R^2^ > 0.999) for a five-point plot were used to determine the concentration of polyphenols. The mobile phase consisted of 95/5 (*v*/*v*) ammonium acetate, 1 mM in water and acetonitrile, isocratic elution, and mobile phase flow rate of 1 mL/min. The mass spectrometer operated in negative mode and nitrogen was used as a nebulising and dry gas. The nebuliser was positioned at 65 psi with the dry gas flow at 12 L /min at 350 °C [45,46]. The determination of polyphenolic acids with higher hydrophilic character (protocatechuic, vanillic, syringic acids) was employed with the same analytical conditions, but using a different binary gradient and compound in MS mode [47]. Analysis of cichoric acid was performed in another stage of work, using the same apparatus previously described, using a different developed procedure of liquid chromatography coupled with mass spectrometry detection. The advantage was a high throughput determination, having a major edge of rapid analysis m/z 293 + m/z 311, which is specific to cichoric acid. The QuantAnalysis 1.7 software (Brucker Daltonics, Darmstadt, Germany) instrumental data system was employed for the quantification of cichoric acid using peak area and the external standard method (R^2^ > 0.999) [48].

### 3.8. Pharmacological Evaluation

#### 3.8.1. Experimental Animals

The experiments were performed on adult male Wistar albino rats, weighing 200–250 g. The animals were bred in the “Iuliu Hațieganu” University of Medicine and Pharmacy Animal Facility. Prior and during to the experiments, animals were housed in proper conditions (12 h night/day cycle, temperatures of 21–22 °C and humidity of 50–55%), with water ad libitum and free access to a standard pellet-based diet (Cantacuzino Institute, Bucharest, Romania). All the animals were sacrificed by cervical dislocation at study completion under general anaesthesia. All treatments that involved animals were in accordance with EU Directive 2010/63/EU. The experimental design was approved by the Institutional Animal Ethical Committee (IAEC) of the “Iuliu Hațieganu” University of Medicine and Pharmacy Cluj-Napoca and by the National Sanitary Veterinary and Food Safety Agency (no. 171/13.07.2019).

#### 3.8.2. Protocols

##### The Evaluation of In Vivo Anti-Inflammatory Effects

For the evaluation of anti-inflammatory effects, 6 groups of animals (n = 5) were used: (1)—the negative control group (CONTROL)—on day 1 received 0.9% saline solution i.m. (6 mL/kg b.w.) and 1 mL orally by gavage, followed by daily administration of 1 mL saline solution, orally; (2)—inflammation group (TURPENTINE)—on day 1 received i.m. turpentine oil (6 mL/kg b.w.) and 0.9% saline solution by gavage, followed by daily administrations of saline solution 0.9% by gavage; (3)—positive control (DICLOFENAC)—on day 1 received i.m. turpentine oil (6 mL/kg b.w.) and sodium diclofenac 10 mg/kg b.w. orally, followed by daily administrations of diclofenac; (4)—TOT 100 group—on day 1 received i.m. turpentine oil (6 mL/kg b.w.) and 1 mL TOT 100 orally, followed by daily administration of 1 mL TOT 100 by gavage; (5)—TOT 50 group—on day 1 received i.m. turpentine oil (6 mL/kg b.w.) and 1 mL orally TOT 50, followed by daily administration of TOT 50 by gavage; (6)—TOT 25 group—on day 1 received i.m. turpentine oil (6 mL/kg b.w.) and 1 mL orally TOT 25, followed by daily administration of 1 mL TOT 25, orally [49,50]. All treatments were performed for seven days.

##### The Evaluation of In Vivo Cardioprotective Effects

In order to evaluate the cardioprotective effects, pre-treatments with TOT were analysed. The animals were divided into 5 groups (n = 5): (1)—negative control (CONTROL); (2)—isoprenaline (ISO); (3)—received TOT 100; (4)—received TOT 50 and (5)—received TOT 25. For seven days the animals received by gavage (orally p.o. 1 mL/day) water, in groups CONTROL and ISO, respectively, the different concentrations of TOT in groups 3, 4, and 5 (p.o. 1 mL/day). Except the CONTROL group, on days 8 and 9 animals received isoprenaline (subcutaneously s.c. 150 mg/kg b.w.) to induce experimental MI [30,51]. On day 10, blood samples were collected by retro-orbital puncture under general anaesthesia induced by a mixture of ketamine (70 mg/kg b.w.) and xilazine (10 mg/kg b.w.) [52]. Serum was separated and stored at −80 °C until the oxidative stress and cardiac markers analysis. Cardiac markers, AST, ALT, and CK-MB, were assessed using commercial kits.

##### The Evaluation of Oxidative Stress Parameters

TOS was assessed using a colorimetric method based on the oxidation of a ferrous ion to a ferric ion in the presence of various oxidant species [53]. The results were expressed in µmol H_2_O_2_ equivalents/L. TAC was measured using a colorimetric assay described by Erel and expressed as mmol Trolox equivalents/L [54]. OSI was calculated as the ratio between TOS and TAC [55]. As a lipid peroxidation marker, MDA was determined using the thiobarbituric acid assay. The MDA serum concentration was expressed as nM/L [56]. The serum NO concentration was assessed using the Griess reaction and expressed as nitrite µM/L [57]. Serum total thiols were expressed as mM GSH/L and were determined using Ellman’s reagent [58].

The NF-kB was determined using a NF-kB ELISA KIT, (ER1186, Fine Biotech, and Wuhan, China) according to the manufacturer instructions.

#### 3.8.3. Statistical Analysis

The statistical analysis was performed using Excel and Statistica 12.0 software. The results are expressed as means ± standard deviation. The data were compared by using a one-way analysis of variance (ANOVA) test and post hoc Bonferroni–Holm test. The correlation between the parameters of the same group was assessed by Pearson’s coefficient (r) in accordance to the Colton scale. The level of significance was established at *p* ˂ 0.05. Multivariate analysis of the parameters was performed using Principal Component Analysis (PCA). On the PCA correlation circle when two vectors are close, forming a small angle, the two variables are positively correlated, if they meet each other at 90°, they are not likely to be correlated, when they diverge and form a large angle (close to 180°), they are negative correlated [21].

## 4. Conclusions

The present study was focused on the common dandelion, *T. officinale*, harvested from the Romanian spontaneous flora. The polyphenolic composition revealed cichoric acid identified in the largest amount, but others phenolic acids (protocatechuic, vanillic, syringic, and ferulic acids), and also flavonoids (rutin, quercitrin, luteolin, and apigenin) were present.

The tested extract (TOT) had a good influence on serum oxidative markers in rat turpentine-induced inflammation model and also in rat ISO-induced MI (TOS, OSI, TAC, MDA, SH, NOx, 513 NF-κB). The pharmacological effects are due to the active principles from the extract; there were identified polyphenols, but also other compounds (e.g., terpenes) that may be present in the tincture. These compounds determined an anti-inflammatory effect in the tested model by lowering the oxidative stress parameters involved in the pro-inflammatory cytokines signalling, based on antioxidant mechanism. The cardioprotective effect of the dandelion tincture was evaluated by the decreasing of the serum cardiac injury enzymes (AST and CK-MB), but also by the diminution of the serum oxidative stress markers levels, increased by isoprenaline in induced MI (TOS, OSI, NOx, MDA).Through our results, we aim to set a basis for further pharmacological inquiries, to extend indigenous medicinal plants uses to new therapeutic directions, combining safety and efficacy.

## Figures and Tables

**Table 1:** The polyphenols content of TOT.

Extract	TPC (mg GAE/g d.w.)	TFC (mg RE/g d.w.)	TCADC (mg CAE/g d.w.)
TOT	26.75 ± 0.73	6.28 ± 0.32	16.74 ± 0.80

Note: Values are expressed as mean of 3 determinations ± SD. GAE—gallic acid equivalents, RE—rutin equivalents, CAE—caffeic acid derivates.

**Table 2:** Phenolic compounds identified in TOT by HPLC-UV-MS.

Polyphenols	[M-H]^−^	Retention Time (min) Rt ± SD	TOT (µg/g d.w.)
Protocatechuic acid	153	2.80 ± 0.05	9.20 ± 0.09
Vanillic acid	167	6.70 ± 0.07	2.00 ± 0.02
Syringic acid	197	8.40 ± 0.09	0.90 ± 0.01
Ferulic acid	193	12.80 ± 0.10	53.60 ± 0.37
Cichoric acid	473	1.12 ± 0.01 *	12,124.89 ± 76.38
Rutin	609	20.20 ± 0.15	14.51 ± 0.10
Quercitrin	447	23.64 ± 0.13	26.08 ± 0.22
Luteolin	285	29.10 ± 0.19	44.08 ± 0.30
Apigenin	269	33.10 ± 0.15	5.79 ± 0.05

Note: Values are the mean ± SD (n = 3). * Determined by a different method characterised by different experimental conditions.

**Table 3:** Antioxidant activity of TOT.

Sample	DPPH· EC50 (µg/mL)	FRAP (µM TE/g)
TOT	165.93 ± 6.94	52.49 ± 1.57

Note: Values are expressed as mean of 3 determinations ± SD.

**Table 4:** Serum oxidative stress markers in rat turpentine-induced inflammation model.

GROUPS	TOS (µM H_2_O_2_ E/L)	OSI	TAC (mM TE/L)	NOx (µM/L)	MDA (nM/L)	SH (mM/L)	NF-κB (ng/mL)
CONTROL	5.13 ± 0.84	4.70 ± 0.77	1.0901 ± 0.001	32.67 ± 2.38	1.91 ± 0.19	0.52 ± 0.05	2.2 ± 0.22
INFLAMM	8.55 ^b^ ± 0.73	8.54 ^b^ ± 0.66	1.0873 ± 0.001	45.34 ^b^ ± 3.53	3.00 ^b^ ±0.21	0.25 ^b^ ± 0.02	4.17 ^a^ ± 0.99
DICLOFENAC	7.84 ± 0.35	7.84 ± 0.32	1.0870 ± 0.000	41.48 ± 2.11	2.94 ± 0.39	0.31 ± 0.04	2.41 ^f^ ± 0.32
TOT 100	4.92 ^e^ ± 0.24	4.92 ^e^ ± 0.22	1.0871 ± 0.001	37.59 ^g^ ± 5.43	3.20 ^g^ ± 0.66	0.20 ^d^ ± 0.02	2.92 ^cg^ ± 0.60
TOT 50	5.15 ^e^ ± 0.72	5.15 ^e^ ± 0.67	1.0878 ± 0.001	37.00 ^g^ ± 3.89	2.91 ^g^ ± 0.39	0.24 ± 0.02	3.64 ± 0.51
TOT 25	4.63 ^e^ ± 0.30	4.63 ^e^ ± 0.27	1.0886 ± 0.000	30.32 ^d^ ± 7.13	3.06 ^g^ ± 0.72	0.27 ^g^ ± 0.11	3.52 ± 0.37

Note: Values are expressed as mean ± SD (n = 5). ^a^ *p* < 0.05, ^b^ *p* < 0.001 versus CONTROL; ^c^ *p* < 0.05, ^d^ *p* < 0.01, ^e^ *p* < 0.001 versus INFLAMM. ^f^ *p* < 0.05 versus INFLAMM. ^g^ *p* > 0.05 versus DICLOFENAC. TOS—total oxidative status, OSI—oxidative stress index, TAC—total antioxidant capacity, Nox—total levels of nitrites and nitrates, MDA—malondialdehyde, SH—total levels of thiols, NF-kB—Nuclear factor kappa-light-chain-enhancer of activated B cells, CONTROL—healthy animals group, INFLAMM group—negative control, DICLOFENAC group—positive control.

**Table 5:** Serum oxidative stress markers in rat isoprenaline (ISO)-induced MI.

GROUPS	TOS (µM H_2_O_2_ E/L)	OSI	TAC (mM TE/L)	NOx (µM/L)	MDA (nM/L)	SH (mM/L)	NF-κB (ng/mL)
CONTROL	5.13 ± 0.84	4.70 ± 0.77	1.0901 ± 0.001	32.67 ± 2.38	1.91 ± 0.19	0.52 ± 0.05	2.2 ± 0.22
ISO	7.43 ^b^ ± 0.11	6.83 ^b^ ± 0.10	1.0876 ± 0.00	45.51 ^b^ ± 0.37	3.41 ^b^ ± 024	0.39 ^b^ ± 0.01	3.42 ^a^ ± 0.59
TOT 100	4.50 ^e^ ± 0.12	4.13 ^e^ ± 0.11	1.0886 ± 0.00	36.49 ± 5.90	2.70 ± 0.20	0.26 ^d^ ± 0.02	2.24 ± 0.50
TOT 50	4.40 ^e^ ±0.12	4.05 ^e^ ± 0.11	1.0873 ± 0.00	34.14 ^c^ ± 3.56	2.38 ^d^ ± 0.14	0.29 ^d^ ± 0.02	1.35 ^c^ ± 0.27
TOT 25	4.19 ^e^ ± 0.13	3.85 ^e^ ± 0.12	1.0880 ± 0.00	30.55 ^e^ ± 3.28	3.26 ± 0.14	0.29 ^d^ ± 0.02	1.05 ^c^ ± 0.18

Note: Values are expressed as mean ± SD (n = 5). ^a^ *p* < 0.01, ^b^ *p* < 0.001 versus CONTROL; ^c^ *p* < 0.05, ^d^ *p* < 0.01, ^e^ *p* < 0.001 versus ISO. TOS—total oxidative status, OSI—oxidative stress index, TAC—total antioxidant capacity, NOx—total levels of nitrites and nitrates, MDA—malondialdehyde, SH—total levels of thiols, NF-kB—Nuclear factor kappa-light-chain-enhancer of activated B cells, CONTROL—healthy animals group, ISO—negative control.

**Table 6:** Serum cardiac injury markers in rat isoprenaline-induced MI.

GROUPS	AST (UI/L)	ALT (UI/L)	CK-MB (UI/L)
CONTROL	35.32 ± 4.89	29.10 ± 4.12	7.26 ± 1.02
ISO	30.94 ± 8.35	40.04 ^a^ ± 7.29	12.11 ^b^ ± 1.08
TOT 100	26.45 ^c^ ± 1.08	24.77 ± 0.55	8.11 ± 1.51
TOT 50	32.52 ± 2.65	26.31 ± 1.22	7.92 ^c^ ± 1.16
TOT 25	30.32 ± 3.53	26.36 ± 2.97	8.47 ± 0.95

Note: Values are expressed as mean ± SD (n = 5). ^a^ *p* < 0.05, ^b^ *p* < 0.001 versus CONTROL; ^c^ *p* < 0.05, versus ISO. AST—aspartate transaminase, ALT—Alanine transaminase, CK–MB—Creatin kinase isoenzyme MB.

## Data Availability

Data is contained within the article and supplementary material.

## Supplementary Materials

The following supporting information can be downloaded at: https://www.mdpi.com/article/10.3390/ph16030358/s1, Figure S1: Chromatogram for 1. ferulic acid, 2. rutin, 3. quercitrin, 4. luteolin, 5. apigenin; Figure S2. Chromatogram for cichoric acid; Figure S3. Chromatogram for protocatechuic, vanilic, syringic acids; Table S1. Pearson Correlations Antiinflammatory activity Taraxaci herba Negative Control; Table S2. Pearson Correlations Antiinflammatory activity Taraxaci herba Positive Control; Table S3. Pearson Correlations Antiinflammatory activity Taraxaci herba TOT; Table S4. Pearson Correlations Antiinflammatory activity Taraxaci herba TOT 1:2; Table S5. Pearson Correlations Antiinflammatory activity Taraxaci herba TOT 1:3; Table S6. Pearson Correlations Cardioprotective activity Taraxaci herba Negative Control; Table S7. Pearson Correlations Cardioprotective activity Taraxaci herba TOT; Table S8. Pearson Correlations Cardioprotective activity Taraxaci herba TOT 1:2; Table S9. Pearson Correlations Cardioprotective activity Taraxaci herba TOT 1:3.
