# Supplementary material for: New Approaches on the Anti-Inflammatory and Cardioprotective Properties of Taraxacum officinale Tincture
Source: Pharmaceuticals (Basel). 2023 Feb 26;16(3):358. doi: 10.3390/ph16030358 (PMC10053582; doi:10.3390/ph16030358)
Supplement: Supplementary file 1 [file pharmaceuticals-16-00358-s001.zip › pharmaceuticals-2201593-supplementary.pdf]

New approaches on the Anti-inflammatory and Cardioprotective properties of *Taraxacum officinale* tincture

Alexandra Epure [1,†], Alina E. Pârvu [2,†], Laurian Vlase [3,*], Daniela Benedec [1], Daniela Hanganu [1], Ovidiu Oniga [4], Ana-Maria Vlase[5], Irina Ielciu[5], Anca Toiu[1] and Ilioara Oniga[1]

1 Department of Pharmacognosy, Faculty of Pharmacy, "Iuliu Hațieganu" University of Medicine and Pharmacy, 8 V. Babeș Street, 400012 Cluj-Napoca, Romania
2 Department of Physiopathology, Faculty of Medicine, "Iuliu Hațieganu" University of Medicine and Pharmacy, 8 V. Babeș Street, 400012 Cluj-Napoca, Romania
3 Department of Pharmaceutical Technology and Biopharmacy, "Iuliu Hațieganu" University of Medicine and Pharmacy, 8 V. Babeș Street, 400012 Cluj-Napoca, Romania
4 Department of Pharmaceutical Chemistry, Faculty of Pharmacy, "Iuliu Hatieganu" University of Medicine and Pharmacy, 8 V. Babeș Street, 400012 Cluj-Napoca, Romania
5 Department of Pharmaceutical Botany, Faculty of Pharmacy, "Iuliu Hațieganu" University of Medicine and Pharmacy, 23 Gheorghe Marinescu Street, 400337 Cluj-Napoca, Romania
* Correspondence: laurian.vlase@umfcluj.ro
† These authors contributed equally to this work.

Supporting information:

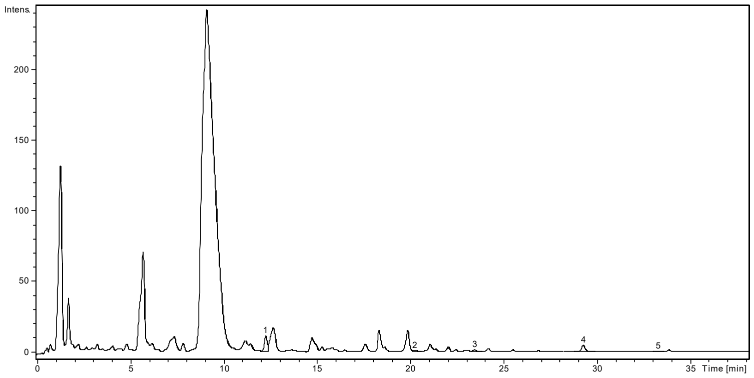

Figure S1. Chromatogram for 1. ferulic acid, 2. rutin, 3. quercitrin, 4. luteolin, 5. apigenin.

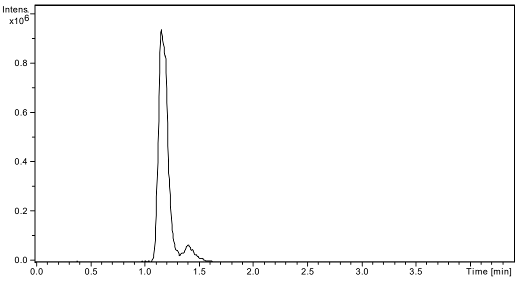

Figure S2. Chromatogram for cichoric acid.

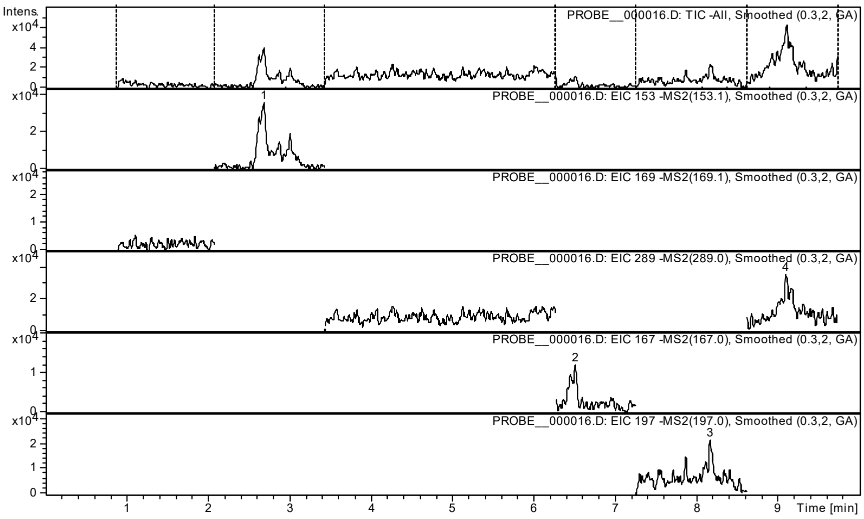

Figure S3. Chromatogram for protocatechuic, vanilic, syringic acids*:

*Polyphenolic carboxylic acids chromatogram, from up to down (Total ion chromatogram, protocatechuic acid extracted ion chromatogram, gallic acid extracted ion chromatogram, catechin and epicatechin extracted ion chromatogram, vanillic acid extracted ion chromatogram, syringic acid extracted ion chromatogram.

Table S1. Pearson Correlations Antiinflammatory activity *Taraxaci herba* Negative Control.

	Taraxaci herba Turpentine Correlations Inflammation						
Variable	TAC	TOS	OSI	NOx	MDA	SH	NF-kB
TAC	1,000000	0,854948	0,850718	0,503524	-0,572217	0,643183	-0,010767
TOS	0,854948	1,000000	0,999967	0,859487	-0,292792	0,615028	0,418387
OSI	0,850718	0,999967	1,000000	0,863017	-0,287744	0,613354	0,424069
NOx	0,503524	0,859487	0,863017	1,000000	0,099250	0,285352	0,788442
MDA	-0,572217	-0,292792	-0,287744	0,099250	1,000000	-0,160190	0,680722
SH	0,643183	0,615028	0,613354	0,285352	-0,160190	1,000000	0,012288
NF-kB	-0,010767	0,418387	0,424069	0,788442	0,680722	0,012288	1,000000

Table S2. Pearson Correlations Antiinflammatory activity *Taraxaci herba* Positive Control.

Variable	Taraxaci herba Diclofenac Correlations Inflammation						
	TAC	TOS	OSI	NOx	MDA	SH	NF-kB
TAC	1,000000	-0,025694	-0,034133	-0,362166	0,257614	0,411942	-0,666249
TOS	-0,025694	1,000000	0,999964	-0,825306	-0,846252	0,713183	0,077848
OSI	-0,034133	0,999964	1,000000	-0,822013	-0,848203	0,709509	0,083417
NOx	-0,362166	-0,825306	-0,822013	1,000000	0,724246	-0,901328	-0,002899
MDA	0,257614	-0,846252	-0,848203	0,724246	1,000000	-0,765594	-0,130058
SH	0,411942	0,713183	0,709509	-0,901328	-0,765594	1,000000	-0,309006
NF-kB	-0,666249	0,077848	0,083417	-0,002899	-0,130058	-0,309006	1,000000

Table S3. Pearson Correlations Antiinflammatory activity *Taraxaci herba* TOT.

Variable	Taraxaci herba tincture Correlations Inflammation						
	TAC	TOS	OSI	NOx	MDA	SH	NF-kB
TAC	1,000000	-0,123647	-0,137381	0,367779	-0,345098	0,551825	0,398581
TOS	-0,123647	1,000000	0,999904	0,728739	0,253151	-0,486684	-0,554169
OSI	-0,137381	0,999904	1,000000	0,722376	0,257680	-0,493654	-0,558548
NOx	0,367779	0,728739	0,722376	1,000000	0,149642	-0,486342	-0,039186
MDA	-0,345098	0,253151	0,257680	0,149642	1,000000	-0,527791	0,413117
SH	0,551825	-0,486684	-0,493654	-0,486342	-0,527791	1,000000	0,071380
NF-kB	0,398581	-0,554169	-0,558548	-0,039186	0,413117	0,071380	1,000000

Table S4. Pearson Correlations Antiinflammatory activity *Taraxaci herba* TOT 1:2.

Variable	Taraxaci herba tincture 1:2 Correlations Inflammation						
	TAC	TOS	OSI	NOx	MDA	SH	NF-kB
TAC	1,000000	-0,734223	-0,736311	-0,185606	0,137542	0,056121	-0,986549
TOS	-0,734223	1,000000	0,999995	0,647314	0,556437	0,620144	0,807499
OSI	-0,736311	0,999995	1,000000	0,645746	0,554039	0,617734	0,809239
NOx	-0,185606	0,647314	0,645746	1,000000	0,571844	0,861096	0,343681
MDA	0,137542	0,556437	0,554039	0,571844	1,000000	0,905446	-0,039635
SH	0,056121	0,620144	0,617734	0,861096	0,905446	1,000000	0,087640
NF-kB	-0,986549	0,807499	0,809239	0,343681	-0,039635	0,087640	1,000000

Table S5. Pearson Correlations Antiinflammatory activity *Taraxaci herba* TOT 1:3.

Variable	Taraxaci herba tincture 1:3 Correlation Inflammation						
	TAC	TOS	OSI	NOx	MDA	SH	NF-kB
TAC	1,000000	-0,394249	-0,399240	-0,315488	-0,163331	0,156687	-0,311629
TOS	-0,394249	1,000000	0,999985	-0,630042	-0,140016	0,603095	0,253519
OSI	-0,399240	0,999985	1,000000	-0,626748	-0,138680	0,600676	0,254856
NOx	-0,315488	-0,630042	-0,626748	1,000000	0,238350	-0,386539	-0,449871
MDA	-0,163331	-0,140016	-0,138680	0,238350	1,000000	-0,567347	-0,226128
SH	0,156687	0,603095	0,600676	-0,386539	-0,567347	1,000000	-0,335928
NF-kB	-0,311629	0,253519	0,254856	-0,449871	-0,226128	-0,335928	1,000000

Table S6. Pearson Correlations Cardioprotective activity *Taraxaci herba* Negative Control.

	Taraxaci herba tincture ISO Correlations Cardiac									
Variable	TAC	TOS	OSI	NOx	MDA	SH	AST	ALT	CK-MB	NF-kB
TAC	1,000000	-0,400437	-0,424774	-0,899243	0,037506	0,394340	0,216034	0,156294	0,431853	0,427152
TOS	-0,400437	1,000000	0,999643	0,048877	-0,925163	-0,746442	-0,973609	-0,961061	-0,996890	0,390002
OSI	-0,424774	0,999643	1,000000	0,074419	-0,915091	-0,748751	-0,968153	-0,954092	-0,997464	0,373065
NOx	-0,899243	0,048877	0,074419	1,000000	0,273368	-0,317673	0,099555	0,225267	-0,102321	-0,775494
MDA	0,037506	-0,925163	-0,915091	0,273368	1,000000	0,724842	0,983600	0,966720	0,917045	-0,519140
SH	0,394340	-0,746442	-0,748751	-0,317673	0,724842	1,000000	0,790371	0,613215	0,794031	0,212470
AST	0,216034	-0,973609	-0,968153	0,099555	0,983600	0,790371	1,000000	0,967923	0,972736	-0,416065
ALT	0,156294	-0,961061	-0,954092	0,225267	0,966720	0,613215	0,967923	1,000000	0,940820	-0,612738
CK-MB	0,431853	-0,996890	-0,997464	-0,102321	0,917045	0,794031	0,972736	0,940820	1,000000	-0,319333
NF-kB	0,427152	0,390002	0,373065	-0,775494	-0,519140	0,212470	-0,416065	-0,612738	-0,319333	1,000000

Table S7. Pearson Correlations Cardioprotective activity *Taraxaci herba* TOT.

	Taraxaci herba tincture Correlations Cardiac									
Variable	TAC	TOS	OSI	NOx	MDA	SH	AST	ALT	CK-MB	NF-kB
TAC	1,000000	0,388952	0,369135	-0,383195	-0,077434	-0,661272	0,278550	-0,351236	0,146771	-0,549003
TOS	0,388952	1,000000	0,999771	-0,730563	-0,535663	-0,342288	0,988953	0,232408	0,760389	0,203102
OSI	0,369135	0,999771	1,000000	-0,727919	-0,538368	-0,330127	0,991168	0,242401	0,763515	0,217471
NOx	-0,383195	-0,730563	-0,727919	1,000000	0,937363	-0,190303	-0,773034	-0,688921	-0,962288	-0,549080
MDA	-0,077434	-0,535663	-0,538368	0,937363	1,000000	-0,517776	-0,623784	-0,897044	-0,953875	-0,790748
SH	-0,661272	-0,342288	-0,330127	-0,190303	-0,517776	1,000000	-0,203015	0,825268	0,300698	0,844537
AST	0,278550	0,988953	0,991168	-0,773034	-0,623784	-0,203015	1,000000	0,364020	0,829309	0,344959
ALT	-0,351236	0,232408	0,242401	-0,688921	-0,897044	0,825268	0,364020	1,000000	0,786562	0,968830
CK-MB	0,146771	0,760389	0,763515	-0,962288	-0,953875	0,300698	0,829309	0,786562	1,000000	0,706808
NF-kB	-0,549003	0,203102	0,217471	-0,549080	-0,790748	0,844537	0,344959	0,968830	0,706808	1,000000

Table S8. Pearson Correlations Cardioprotective activity *Taraxaci herba* TOT 1:2.

	Taraxaci herba tincture 1:2 Correlations Cardiac									
Variable	TAC	TOS	OSI	NOx	MDA	SH	AST	ALT	CK-MB	NF-kB
TAC	1,000000	0,421707	0,410897	0,286944	0,840031	-0,487980	-0,289533	-0,382655	-0,161882	0,081875
TOS	0,421707	1,000000	0,999929	-0,191683	0,427630	-0,077709	0,573363	0,477676	-0,598953	-0,851086
OSI	0,410897	0,999929	1,000000	-0,196670	0,418845	-0,071882	0,580187	0,485328	-0,599850	-0,856890
NOx	0,286944	-0,191683	-0,196670	1,000000	0,692566	-0,686442	-0,672182	-0,851013	0,370650	0,474772
MDA	0,840031	0,427630	0,418845	0,692566	1,000000	-0,700501	-0,378184	-0,564492	-0,050372	0,057679
SH	-0,487980	-0,077709	-0,071882	-0,686442	-0,700501	1,000000	0,742783	0,737873	-0,635324	-0,149719
AST	-0,289533	0,573363	0,580187	-0,672182	-0,378184	0,742783	1,000000	0,948915	-0,824683	-0,752311
ALT	-0,382655	0,477676	0,485328	-0,851013	-0,564492	0,737873	0,948915	1,000000	-0,647469	-0,758542
CK-MB	-0,161882	-0,598953	-0,599850	0,370650	-0,050372	-0,635324	-0,824683	-0,647469	1,000000	0,474574
NF-kB	0,081875	-0,851086	-0,856890	0,474772	0,057679	-0,149719	-0,752311	-0,758542	0,474574	1,000000

Table S9. Pearson Correlations Cardioprotective activity *Taraxaci herba* TOT 1:3.

	Taraxaci herba tincture 1:3 Correlations Cardiac									
Variable	TAC	TOS	OSI	NOx	MDA	SH	AST	ALT	CK-MB	NF-kB
TAC	1,000000	-0,104152	-0,114063	-0,483875	0,052030	0,351948	0,432055	0,350777	-0,570173	-0,680548
TOS	-0,104152	1,000000	0,999950	0,166876	0,420464	0,370261	0,717515	0,611981	-0,671813	0,589868
OSI	-0,114063	0,999950	1,000000	0,171716	0,419856	0,366183	0,712271	0,607713	-0,665164	0,595775
NOx	-0,483875	0,166876	0,171716	1,000000	0,712801	-0,851471	-0,526932	-0,669708	0,270645	0,134491
MDA	0,052030	0,420464	0,419856	0,712801	1,000000	-0,454008	-0,051095	-0,186278	-0,118082	-0,227153
SH	0,351948	0,370261	0,366183	-0,851471	-0,454008	1,000000	0,857886	0,953416	-0,564001	0,203232
AST	0,432055	0,717515	0,712271	-0,526932	-0,051095	0,857886	1,000000	0,957046	-0,877806	0,273237
ALT	0,350777	0,611981	0,607713	-0,669708	-0,186278	0,953416	0,957046	1,000000	-0,705648	0,255221
CK-MB	-0,570173	-0,671813	-0,665164	0,270645	-0,118082	-0,564001	-0,877806	-0,705648	1,000000	-0,172485
NF-kB	-0,680548	0,589868	0,595775	0,134491	-0,227153	0,203232	0,273237	0,255221	-0,172485	1,000000
